# Prediabetes Is Associated with an Increased Risk of Testosterone Deficiency, Independent of Obesity and Metabolic Syndrome

**DOI:** 10.1371/journal.pone.0074173

**Published:** 2013-09-12

**Authors:** Chen-Hsun Ho, Hong-Jeng Yu, Chih-Yuan Wang, Fu-Shan Jaw, Ju-Ton Hsieh, Wan-Chung Liao, Yeong-Shiau Pu, Shih-Ping Liu

**Affiliations:** 1 Graduate Institute of Clinical Medicine, College of Medicine, National Taiwan University, Taipei, Taiwan; 2 Department of Urology, National Taiwan University Hospital and College of Medicine, Taipei, Taiwan; 3 Department of Internal Medicine, National Taiwan University Hospital and College of Medicine, Taipei, Taiwan; 4 Institute of Biomedical Engineering, National Taiwan University, Taipei, Taiwan; 5 Health Management Center, National Taiwan University Hospital, Taipei, Taiwan; University of Warwick – Medical School, United Kingdom

## Abstract

**Objective:**

The association between type 2 diabetes and low testosterone has been well recognized. However, testosterone levels in men with prediabetes have been rarely reported. We aimed to investigate whether prediabetes was associated with an increased risk of testosterone deficiency.

**Methods:**

This study included 1,306 men whose sex hormones was measured during a medical examination. Serum total testosterone and sex hormone-binding globulin were measured; free and bioavailable testosterone concentrations were calculated by Vermeulen’s formula. Prediabetes was defined by impaired fasting glucose (IFG), impaired postprandial glucose (IPG), or glycated hemoglobin (HbA1c) 5.7%-6.4%. Logistic regression was performed to obtain the odds ratios (OR) for subnormal total testosterone (<300 ng/dL) or free testosterone (<6 ng/dL) in prediabetic and diabetic men compared with normoglycemic individuals, while adjusting for age, BMI, waist circumference, and metabolic syndrome (MetS).

**Results:**

Normoglycemia, prediabetes, and diabetes were diagnosed in 577 (44.2%), 543 (41.6%), and 186 (14.2%) men, respectively. Prediabetes was associated with an increased risk of subnormal total testosterone compared to normoglycemic individuals (age-adjusted OR=1.87; 95%CI=1.38-2.54). The risk remained significant in all multivariate analyses. After adjusting for MetS, the OR in prediabetic men equals that of diabetic patients (1.49 versus 1.50). IFG, IPG, and HbA1c 5.7%-6.4% were all associated with an increased risk of testosterone deficiency, with different levels of significance in multivariate analyses. However, neither prediabetes nor diabetes was associated with subnormal free testosterone in multivariate analyses.

**Conclusions:**

Prediabetes is associated with an increased risk of testosterone deficiency, independent of obesity and MetS. After adjusting for MetS, the risk equals that of diabetes. Our data suggest that testosterone should be measured routinely in men with prediabetes.

## Introduction

Type 2 diabetes is associated with testosterone deficiency (TD), as cross-sectional studies have shown approximately 25% to 40% of diabetic men have low testosterone [1-3] and longitudinal studies have demonstrated that men with low testosterone are at a greater risk of future development of type 2 diabetes [4-7]. While the mechanisms are not fully understood, current evidence suggests that the causative relationship between TD and diabetes might be bidirectional, or even multidirectional and interrelated with obesity, metabolic syndrome (MetS), sex hormone-binding globulins (SHBG), and other factors [8,9].

Prediabetes is a condition in which blood glucose level is higher than normal but does not reach the level of diabetes. Most diabetes organizations define prediabetes by impaired fasting glucose (IFG) and impaired glucose tolerance (IGT), while the American Diabetes Association (ADA) proposes that a glycated hemoglobin (HbA1c) of 5.7% to 6.4% should be also considered one of the criteria [10]. Prediabetes is characterized by increased insulin resistance and β-cell dysfunction, and is considered to precede the development of type 2 diabetes [11]. Data from non-diabetic men have revealed an inverse association between insulin resistance and testosterone concentrations [12-14]. This raised the question whether prediabetes, a state of increased insulin resistance, is also associated with low testosterone. However, few studies have investigated sex hormone levels in men with prediabetes, and the risk of TD in men with prediabetes has not been reported.

The present study aimed to investigate whether men with prediabetes were at an increased the risk of TD. Furthermore, as prediabetes is closely linked to obesity and MetS, both of which are associated with TD [8], we also explored whether the relationship between prediabetes and TD, if present, was independent of these factors.

## Materials and Methods

### Study design and subjects

This is a cross-sectional study. We obtained the data from the database of Health Management Center, National Taiwan University Hospital. In 2009, a total of 1,339 men received sex hormone measurement as part of their medical examination. We excluded those who received testosterone supplement (n=3) or received androgen deprivation therapy for prostate cancer (n=7) and those whose data were incompletely recorded (n=23). The remaining 1,306 constituted the study subjects of the current study. The study protocol was approved by the institutional review board (IRB) of National Taiwan University Hospital (201207058RIC). The IRB waived the need for consent, since all data were de-identified and the investigators could not recognize the identity of any subject.

### Collection of basic data

All participants completed a self-administered questionnaire to collect their basic demographic data and medical histories. All subjects were then interviewed by an internal medicine physician, and a detailed physical examination was performed.

### Laboratory tests and sex hormone parameters

Two blood samples were collected from each subject: the first sample was collected after an overnight fast between 8 am and 10 am, and was used to measure fasting blood glucose, sex hormones, and other biochemical data; the second blood sample was collected two hours after a standard lunch and was used to measure the postprandial glucose. Total testosterone and SHBG were measured by chemiluminescent microparticle immunoassay using the Architect Testosterone and SHBG Reagent kits (Abbott Laboratories, Chicago, IL), respectively. Free testosterone was calculated by the formula proposed by Vermeulen [15]. The free testosterone concentration calculated by this formula correlate well with the concentrations measured by equilibrium dialysis and ammonium sulfate precipitation, respectively [15], and the formula has been widely adopted by other investigators [1,2,13]. Low total testosterone was defined by total testosterone <300 ng/dL [16-18], and low free testosterone was defined by free testosterone <6 ng/dL.

### Diagnoses of prediabetes, diabetes, and metabolic syndrome

Prediabetes was diagnosed if any of the following criteria was met: 1) fasting glucose 100-125 mg/dL (IFG), 2) two-hour postprandial glucose 140-199 mg/dL (IPG), or 3) HbA1c 5.7%-6.4%. The definition was adopted from the recommendations of the ADA [10], with the exception that two-hour postprandial glucose was used to replace a standard oral glucose tolerance test (OGTT) [19,20]. Diabetes was diagnosed if the patient had a prior history of diabetes or if the glycemic variables reached the criteria of diabetes: fasting glucose ≥126 mg/dL, two-hour postprandial glucose ≥200 mg/dL, or HbA1c ≥6.5% [10]. The diagnosis of MetS was based on the modified ATP III criteria for Asians [21], and one was considered to have MetS if any three of the following were met: 1) waist circumference >90 cm, 2) triglycerides >150 mg/dL, 3) high-density lipoprotein cholesterol <40 mg/dL, 4) blood pressure >130/85 mmHg, and 5) fasting glucose >100 mg/dL.

### Statistical analyses

Continuous data are presented as the mean ± standard deviation (SD), and categorical data are presented as count and percentage (%). Logistic regression was performed to obtain the odds ratios for TD in men with prediabetes and diabetes compared with those with normoglycemia. Five statistical models were used for multivariate analyses: Model 1, adjusted for age; Model 2, adjusted for age and body mass index (BMI); Model 3, adjusted for age and waist circumference; Model 4, adjusted for age and the number of MetS components; Model 5, adjusted for age and MetS. Multiple linear regression was performed to assess the association between total and free testosterone and prediabetes or diabetes. All statistical assessments were two-tailed, and a p-value of <0.05 was considered significant. All statistical procedures were performed using SPSS 17.0 (SPSS Inc., Chicago, IL).

## Results

Among the 1,306 male participants, 577 (44.1%) were normoglycemic, 543 (41.5%) were prediabetic, and 186 (14.4%) were diabetic. The characteristics of the three groups are listed in Table 1. The mean ages of the three groups were 52.6 ± 8.8, 55.8 ± 8.5, and 59.7 ± 7.9 years (P<0.001); the mean BMIs were 24.4 ± 3.0, 25.2 ± 3.1, and 25.6 ± 3.3 kg/m^2^ (P<0.001); the mean waist circumferences were 86.6 ± 7.4, 89.2 ± 7.5, and 90.6 ± 8.7 cm (P<0.001). Metabolic syndrome was present in 12.9%, 35.4%, and 60.5% of the patients (P<0.001), and the mean number of MetS components was 1.1 ± 1.1, 2.0 ± 1.3, and 2.8 ± 1.3 (P<0.001). The mean testosterone concentration and the prevalence of subnormal testosterone are listed in Table 2.

**Table 1:** Characteristics of the 1,306 male subjects.

	NGT	Prediabetes	Diabetes
	n=577	n=543	n=186
Age (years)	52.6 ± 8.6	55.8 ± 8.5	59.7 ± 7.9
Age range (years)	51.8-53.3	55.1-56.6	58.6-60.9
BMI (kg/m^2^)	24.4 ± 3.0	25.2 ± 3.1	25.6 ± 3.3
BMI range (kg/m2)	24.2-24.6	25.0-25.5	25.1-26.0
Waist (cm)	86.6 ± 7.4	89.2 ± 7.5	90.6 ± 8.7
Waist >=90 cm (%)	32.9%	47.7%	50.3%
Fasting glucose (mg/dL)	89.3 ± 5.3	98.2 ± 8.9	123.4 ± 31.6
Postprandial glucose (mg/dL)	99.5 ± 18.9	127.9 ± 31.2	203.0 ± 66.0
HbA1c (%)	5.34 ± 0.23	5.71 ± 0.28	6.73 ± 1.09
HbA1c range (%)	5.32-5.36	5.69-5.74	6.57-6.88
Triglyceride (mg/dL)	122.5 ± 67.7	143.9 ± 77.5	158.0 ± 105.2
TG >=150 mg/dL (%)	26.2%	38.3%	41.4%
Cholesterol (mg/dL)	202.4 ± 33.3	208.2 ± 33.8	195.2 ± 41.0
LDL (mg/dL)	119.3 ± 30.1	124.5 ± 30.7	113.4 ± 36.5
HDL (mg/dL)	47.4 ± 10.3	45.7 ± 11.1	44.1 ± 10.6
HDL<40 mg/dL (%)	22.9%	29.1%	40.3%
Systolic pressure (mmHg)	117.4 ±17.0	122.3 ± 14.9	124.3 ± 14.7
Diastolic pressure (mmHg)	69.5 ± 11.5	72.1 ± 10.0	71.8 ± 9.6
Hypertension (%)	29.3%	44.9%	60.8%
Num. of MetS components	1.1 ± 1.1	2.0 ± 1.3	2.8 ± 1.3
MetS (%)	12.9%	35.4%	60.5%

**Table 2:** Mean testosterone concentrations and prevalence of testosterone deficiency within subgroups.

			**NGT**	**Prediabetes**	**Diabetes**	**Total**	P value
**All**		Case number	577	543	186	1306	
		mean TT, ng/dL	440.5 ± 152.2	397.2 ± 133.6	381.2 ± 132.9	414.1 ± 143.9	<0.001
		TT <300 ng/dL, N(%)	88 (15.3%)	131 (24.1%)	51 (27.4%)	270 (20.7%)	<0.001
		mean FT, ng/dL	8.6 ± 2.6	8.1 ± 2.4	7.7 ± 2.6	8.3 ± 2.6	<0.001
		FT <6 ng/dL, N(%)	77 (13.3%)	97 (17.9%)	37 (19.9%)	211 (16.2%)	0.004
**Age**	50	Case number	216	117	17	350	
		mean TT, ng/dL	438.0 ± 146.7	383.5 ± 123.3	331.2 ± 134.6	414.6 ± 141.9	<0.001
		TT <300 ng/dL, N(%)	38 (17.6%)	32 (27.4%)	8 (47.1%)	78 (22.3%)	0.005
		mean FT, ng/dL	9.1 ± 2.7	8.7 ± 2.5	7.3 ± 2.6	8.9 ± 2.6	0.018
		FT <6 ng/dL, N(%)	21 (9.7%)	13 (11.1%)	5 (29.4%)	39 (11.1%)	0.046
	50-59	Case number	252	251	80	583	
		mean TT, ng/dL	433.6 ± 159.2	395.9 ± 130.5	394.7 ± 146.3	412.0 ± 146.6	0.008
		TT <300 ng/dL, N(%)	38 (15.1%)	58 (23.1%)	21 (26.3%)	117 (20.1%)	0.027
		mean FT, ng/dL	8.4 ± 2.7	8.3 ± 2.4	8.2 ± 3.1	8.3 ± 2.6	0.85
		FT <6 ng/dL, N(%)	39 (15.5%)	44 (17.5%)	14 (17.5%)	97 (16.6%)	0.806
	60	Case number	109	175	89	373	
		mean TT, ng/dL	461.6 ± 145.6	408.3 ± 143.9	378.7 ± 118.1	416.8 ± 141.8	<0.001
		TT <300 ng/dL, N(%)	12 (11%)	41 (23.4%)	22 (24.7%)	75 (20.1%)	0.018
		mean FT, ng/dL	8.0 ± 2.4	7.5 ± 2.3	7.3 ± 2.0	7.6 ± 2.3	0.111
		FT <6 ng/dL, N(%)	17 (15.6%)	40 (22.9%)	18 (20.2%)	75 (20.1%)	0.332
**BMI**	24	Case number	248	182	58	488	
		mean TT, ng/dL	472.1 ± 156.3	437.0 ± 149.0	428.9 ± 155.1	453.9 ± 154.3	0.028
		TT <300 ng/dL, N(%)	24 (9.7%)	30 (16.5%)	9 (15.5%)	63 (12.9%)	0.094
		mean FT, ng/dL	8.7 ± 2.8	8.4 ± 2.8	8.0 ± 3.5	8.5 ± 2.9	0.223
		FT <6 ng/dL, N(%)	34 (13.7%)	32 (17.6%)	15 (25.9%)	81 (16.6%)	0.074
	24-26.9	Case number	229	223	73	525	
		mean TT, ng/dL	426.9 ± 150.3	392.7 ± 126.2	380.8 ± 116.7	405.9 ± 137.1	0.007
		TT <300 ng/dL, N(%)	43 (18.8%)	59 (26.5%)	16 (21.9%)	118 (22.5%)	0.147
		mean FT, ng/dL	8.5 ± 2.6	8.1 ± 2.3	7.9 ± 2.1	8.3 ± 2.4	0.079
		FT <6 ng/dL, N(%)	28 (12.2%)	42 (18.8%)	9 (12.3%)	79 (15%)	0.114
	≧27	Case number	100	138	55	293	
		mean TT, ng/dL	393.4 ± 128.7	352.1 ± 105.9	331.6 ± 109.5	362.3 ± 116.8	0.002
		TT <300 ng/dL, N(%)	21 (21%)	42 (30.4%)	26 (47.3%)	89 (30.4%)	0.003
		mean FT, ng/dL	8.4 ± 2.5	7.8 ± 2.1	7.1 ± 2.1	7.9 ± 2.3	0.003
		FT <6 ng/dL, N(%)	15 (15%)	23 (16.7%)	13 (23.6%)	51 (17.4%)	0.379
**MS**	negative	Case number	500	351	73	924	
		mean TT, ng/dL	451.2 ± 151.5	421.7 ± 143.1	415.3 ± 122.9	437.2 ± 146.9	0.006
		TT <300 ng/dL, N(%)	63 (12.6%)	69 (19.7%)	9 (12.3%)	141 (15.3%)	0.014
		mean FT, ng/dL	8.7 ± 2.7	8.3 ± 2.6	7.8 ± 2.5	8.5 ± 2.6	0.015
		FT <6 ng/dL, N(%)	62 (12.4%)	62 (17.7%)	17 (23.3%)	141 (15.3%)	0.015
	positive	Case number	74	192	112	378	
		mean TT, ng/dL	365.3 ± 132.6	352.5 ± 99.8	359.4 ± 135.5	357.1 ± 117.8	0.707
		TT <300 ng/dL, N(%)	24 (32.4%)	62 (32.3%)	42 (37.5%)	128 (33.9%)	0.625
		mean FT, ng/dL	8.0 ± 2.3	7.7 ± 2.1	7.7 ± 2.7	7.8 ± 2.3	0.662
		FT <6 ng/dL, N(%)	14 (18.9%)	35 (18.2%)	20 (17.9%)	69 (18.3%)	0.983

P value compares continuous variables with t test and categorical variables with chi-square test.

TT, total testosterone; FT, free testosterone; MS, metabolic syndrome; NS, not significant

The age-adjusted odds ratio for subnormal total testosterone was 1.87 (95% confidence interval [CI]: 1.38-2.54) in prediabetic men and 2.38 (95% CI: 1.57-3.60) in diabetic patients (Model 1 in Table 3). The odds ratios in prediabetic men remained significant in multivariate analyses that further adjusted for BMI, waist circumference, the number of MetS components, and MetS (Models 2-5 in Table 3). After adjustment for MetS, the odds ratio for subnormal total testosterone in prediabetic men was 1.49 (95% CI: 1.08-2.06), almost equal to the odds ratio of 1.50 in diabetic patients (Model 5 in Table 3).

**Table 3:** Adjusted odds ratios for total testosterone <300 ng/dL in multivariate analyses.

	Model 1	Model 2	Model 3	Model 4	Model 5
	OR	OR	OR	OR	OR
Normoglycemia	1.00	1.00	1.00	1.00	1.00
Prediabetes	1.87 (1.38,2.54)^+^	1.69 (1.24,2.30)^♯^	1.63 (1.19,2.23)^♯^	1.50 (1.09,2.06)*	1.49 (1.08,2.06)*
Diabetes	2.38 (1.57,3.60)^+^	2.03 (1.33,3.09)^♯^	1.90 (1.24,2.92)^♯^	1.62 (1.05,2.50)*	1.50 (0.96,2.35)
					
FPG (mg/dL)					
<100	1.00	1.00	1.00	1.00	1.00
100-125	1.82 (1.32,2.50)^+^	1.66 (1.20,2.30)^♯^	1.52 (1.09,2.11)*	1.45 (1.03,2.02)*	1.19 (0.83,1.22)
>=126 or known DM	1.99 (1.30,3.03)^♯^	1.71 (1.11,2.64)*	1.61 (1.04,2.49)*	1.40 (0.9,2.18)	1.22 (0.77,1,93)
					
PPG (mg/dL)					
<140	1.00	1.00	1.00	1.00	1.00
140-199	1.46 (1.04,2.07)*	1.42 (1.00,2.02)	1.40 (0.98,1.99)	1.22 (0.85,1.75)	1.23 (0.86,1.75)
>=200 or known DM	1.67 (1.11,2.52)*	1.53 (1.01,2.33)*	1.47 (0.96,2.25)	1.29 (0.85,1.98)	1.16 (0.75,1.78)
					
HbA1c (%)					
<5.7	1.00	1.00	1.00	1.00	1.00
5.7-6.4	1.77 (1.31,2.39)^+^	1.56 (1.15,2.12)^♯^	1.48 (1.08,2.02)*	1.44 (1.05,1.97)*	1.46 (1.07,2.00)*
>=6.5 or known DM	2.19 (1.46,3.30)^+^	1.83 (1.20,2.79)^♯^	1.69 (1.10,2.59)*	1.51 (0.99,2.33)	1.50 (0.91,2.18)

FPG: fasting plasma glucose; PPG: postprandial plasma glucose Model 1: adjusted for age Model 2: adjusted for age and BMI Model 3: adjusted for age and waist circumference Model 4: adjusted for age and numbers of MetS components Model 5: adjusted for age and MetS *:p<0.05; ♯:p<0.01; +:<0.001

The presence of IFG, IPG, and HbA1c 5.7%-6.4% were all significantly associated with an increased risk of TD after adjustment for age (Model 1 in Table 3). In multivariate analyses, HbA1c 5.7%-6.4% remained significant in models that further adjusted for BMI, waist circumference, the number of MetS components, or MetS (Models 2-5 in Table 3). IFG was significant in most multivariate analyses (Models 2 to 4), but was no longer significant after adjustment for MetS (Model 5 in Table 3). However, IPG was not significant after any further adjustments (Models 2-5 in Table 3).

**Figure 1:**
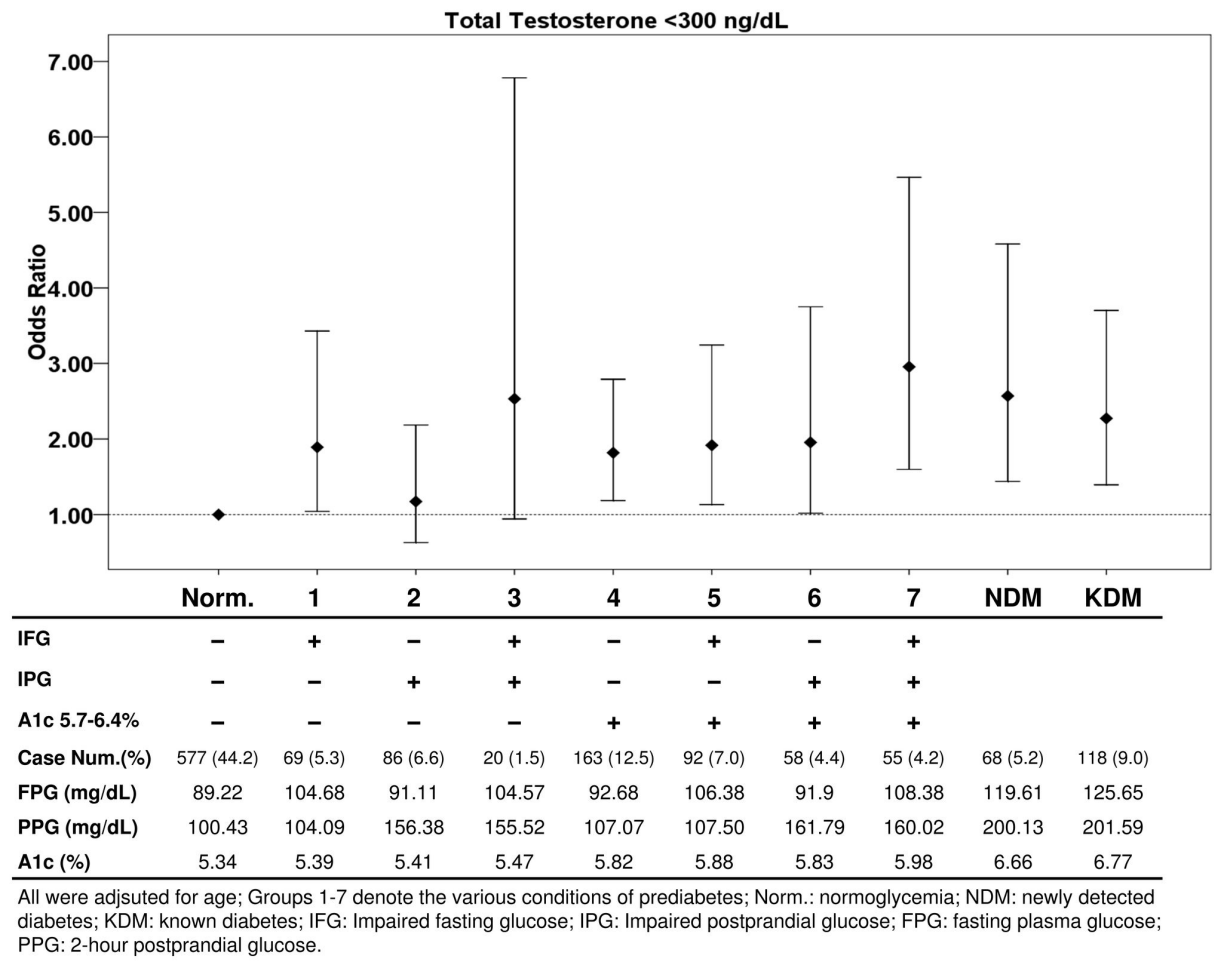
Age-adjusted Odds Ratios for Total Testosterone <300 ng/dL in Various Conditions of Hyperglycemia.

Figure 1 demonstrates the age-adjusted odds ratios for subnormal total testosterone in various conditions of hyperglycemia as classified by fasting glucose, postprandial glucose, and HbA1c. Compared to those with normoglycemia, men with isolated IFG, HbA1c 5.7%-6.4% (with or without elevations in fasting or postprandial glucose), newly detected diabetes, or previously diagnosed diabetes were all at an increased risk for TD.

Table 4 shows the adjusted odds ratios for subnormal free testosterone in various glycemic groups. Neither prediabetes nor diabetes was associated with an increased risk of subnormal free testosterone after adjustment for age, BMI, waist circumference, the number of MetS, or MetS. IFG, IPG, or HbA1c 5.7%-6.4% was not associated with subnormal free testosterone in all multivariate analyses.

**Table 4:** Adjusted odds ratios for free testosterone <6 ng/dL in multivariate analyses.


	Model 1	Model 2	Model 3	Model 4	Model 5
	OR	OR	OR	OR	OR
Normoglycemia	1.00	1.00	1.00	1.00	1.00
Prediabetes	1.27 (0.91,1.77)	1.27 (0.91,1.78)	1.26 (0.90,1.76)	1.20 (0.85,1.68)	1.25 (0.89,1.76)
Diabetes	1.28 (0.81,2.00)	1.28 (0.81,2.01)	1.25 (0.79,1.98)	1.15 (0.72,1.83)	1.23 (0.76,1.99)
FPG (mg/dL)					
<100	1.00	1.00	1.00	1.00	1.00
100-125	1.04 (0.72,1.51)	1.04 (0.71,1.51)	1.01 (0.69,1.47)	0.97 (0.66,1.41)	0.97 (0.65,1.45)
>=126 or known DM	1.07 (0.67,1.71)	1.07 (0.66,1.71)	1.04 (0.65,1.67)	0.96 (0.59,1.55)	0.99 (0.59,1.63)
					
PPG (mg/dL)					
<140	1.00	1.00	1.00	1.00	1.00
140-199	1.41 (0.97,2.04)	1.41 (0.97,2.04)	1.41 (0.97,2.04)	1.34 (0.92,1.95)	1.39 (0.96,2.02)
>=200 or known DM	1.25 (0.80,1.95)	1.24 (0.80,1.95)	1.23 (0.79,1.93)	1.17 (0.74,1.84)	1.21 (0.76,1.92)
					
HbA1c (%)					
<5.7	1.00	1.00	1.00	1.00	1.00
5.7-6.4	1.23 (0.89,1.72)	1.23 (0.88,1.72)	1.21 (0.86,1.69)	1.16 (0.83,1.62)	1.21 (0.86,1.69)
>=6.5 or known DM	1.07 (0.68,1.71)	1.08 (0.67,1.72)	1.05 (0.65,1.68)	0.96 (0.60,1.55)	1.01 (0.62,1.66)

FPG: fasting plasma glucose; PPG: postprandial plasma glucose

Model 1: adjusted for ageModel 2: adjusted for age and BMIModel 3: adjusted for age and waist circumferenceModel 4: adjusted for age and numbers of MetS componentsModel 5: adjusted for age and MetS

The association between testosterone and prediabetes or diabetes was also examined with multiple linear regression (Tables 5 and 6). Both prediabetes and diabetes were significant associated with total testosterone, independent of age, BMI, waist circumference, the number of MetS components, and MetS. The effect of prediabetes on total testosterone concentration was similar to that of diabetes (Model 5 in Table 5). On the contrary, the association between free testosterone and prediabetes was not significant in all multivariate analyses (Table 6).

**Table 5:** Multiple linear regression assessing the association between total testosterone and prediabetes or diabetes.

		Prediabetes				Diabetes	
Adjustment	Beta	95% CI	P		Beta	95% CI	P
Model 1	-0.045	(-0.063, -0.027)	<0.001		-0.068	(-0.094, -0.042)	<0.001
Model 2	-0.036	(-0.054, -0.018)	<0.001		-0.053	(-0.079, -0.028)	<0.001
Model 3	-0.032	(-0.050, -0.014)	<0.001		-0.047	(-0.073, -0.022)	<0.001
Model 4	-0.025	(-0.043, -0.007)	0.005		-0.034	(-0.060, -0.009)	0.009
Model 5	-0.027	(-0.045, -0.008)	0.004		-0.029	(-0.056, -0.001)	0.039

The value of total testosterone was log transformed

The effect of preidabetes or diabetes was compared with normoglycemia

Model 1: adjusted for ageModel 2: adjusted for age and BMIModel 3: adjusted for age and waist circumferenceModel 4: adjusted for age and numbers of MetS componentsModel 5: adjusted for age and MetS

**Table 6:** Multiple linear regression assessing the association between free testosterone and prediabetes or diabetes.

		Prediabetes				Diabetes	
Adjustment	Beta	95% CI	P		Beta	95% CI	P
Model 1	-0.012	(-0.028, 0.004)	NS		-0.024	(-0.047, -0.001)	0.04
Model 2	-0.010	(-0.026, 0.007)	NS		-0.021	(-0.044, 0.003)	NS
Model 3	-0.008	(-0.024, 0.008)	NS		-0.018	(-0.041, 0.006)	NS
Model 4	-0.004	(-0.020, 0.012)	NS		-0.011	(-0.034, 0.013)	NS
Model 5	-0.005	(-0.021, 0.012)	NS		-0.009	(-0.034, 0.015)	NS

The value of free testosterone was log transformed

The effect of preidabetes or diabetes was compared with normoglycemia

Model 1: adjusted for ageModel 2: adjusted for age and BMIModel 3: adjusted for age and waist circumferenceModel 4: adjusted for age and numbers of MetS componentsModel 5: adjusted for age and MetS

## Discussion

In the present study, we aimed to elucidate whether men with prediabetes had a greater risk for TD, while adjusting for obesity and MetS. The major findings are as follows: 1) prediabetes was associated with an increased risk of subnormal total testosterone, and the risk remained significant in multivariate analyses that adjusted for age, BMI, waist circumferences, the number of MetS components, and MetS; 2) after adjustment for MetS, the risk for subnormal total testosterone in men with prediabetes was equal to that in men with diabetes; 3) neither prediabetes nor diabetes was associated with subnormal free testosterone in multivariate analyses.

While there is a substantial body of evidence demonstrating the association between low testosterone and diabetes [1-7], few studies have reported the androgen status of men with prediabetes, which is considered the preceding stage of overt diabetes. In a study including 221 non-diabetic men, total testosterone was inversely associated with fasting glucose level and insulin resistance, and the association was independent of total body fat or abdominal fat [13]. Moreover, the Rancho Bernardo Study evaluated the sex hormone levels in 775 men and showed that men with IFG or IGT had lower total testosterone than those with normal glucose tolerance, after adjustment for age and BMI [22]. More recently, Corona et al. [23]evaluated the impact of IFG on sexual health in a consecutive 3,451 men attending the clinic for sexual dysfunction, and found that IFG increases the risk of severe erectile dysfunction, reduced penile flow, and overt hypogonadism [23]. Our findings in large part concur with the results of the previous studies, but there are some difference and several novel findings. First, the present study adopted a more updated definition of prediabetes, in which an HbA1c of 5.7%-6.4% was incorporated in the criteria of prediabetes and was proved as a significant risk factor for TD. To our knowledge, it is the first study reporting the association between HbA1c and testosterone in non-diabetic population. Second, we made a more comprehensive multivariate analysis, which adjusted for not only age but also several factors associated with obesity and MetS. The risk was reduced but remained significant after adjustment for BMI (a proxy of total body fat) or waist circumference (a proxy of visceral abdominal fat), suggesting a mechanism other than the consumption by fat is involved in the relationship between TD and prediabetes or diabetes. And we also found that men with prediabetes were at virtually the same risk of subnormal total testosterone as men with diabetes after adjustment for MetS. Third, while the Rancho Bernardo Study exclusively enrolled men aged over 55 years (mean age: 71.9 years), the present study included male participants aged 24 to 86 years (mean age: 54.9 years). The association between prediabetes and testosterone TD could therefore be applied to men over a wider age range. As the Endocrine Society recommends routine measurement of testosterone in all men with type 2 diabetes, the substantially increased risk of TD shown in the present study suggests the measurement should be performed earlier, as at the stage of prediabetes.

The definition of prediabetes differs among health organizations. It is generally composed of IFG and IGT, although different cutoff values to define IFG were adopted by the ADA and the World Health Organization (WHO) (100 mg/dL or 110 mg/dL, respectively) [10]. Greater controversy exists over whether HbA1c of 5.7% to 6.4% should be considered as prediabetes. Currently, an intermediate HbA1c range is considered prediabetes by the ADA [10], but not by the WHO. It has to be noted that the selection of glycemic measures and cutoff values to define prediabetes is based on the risk of future development of overt diabetes. However, elevations in fasting and postprandial glucose levels may be caused by different mechanisms and may represent distinct stages of diabetes development [11,24-27]. Prediabetes identified by IFG, IGT, or an intermediate HbA1c range may represent different population and is associated with different features of metabolic derangement [28,29]. Moreover, fasting and postprandial hyperglycemia have been shown to be associated with different levels of risk of disease, such as cardiovascular events and mortality [30,31]. Intrigued by these findings, we further examined whether the risk of TD might differ in men with IFG, IPG, or HbA1c 5.7%-6.4%. Our data show that HbA1c appeared to be a stronger predictor of subnormal total testosterone, and it remained significant across all multivariate analyses. Men with HbA1c 5.7%-6.4%, with or without fasting or postprandial hyperglycemia, were at a significantly increased risk of subnormal total testosterone. IFG was also a reliable predictor in most multivariate adjustments, and loss of significance was only observed after adjustment for MetS. An isolated IFG, even with HbA1c of <5.7%, was significantly associated with an increased risk of subnormal total testosterone. In contrast, compared with fasting glucose or HbA1c levels, postprandial glucose was only weakly associated with TD in the present study. Although postprandial glucose has been used in lieu of a standard OGTT to define prediabetes in the literature [19], the clinical application of postprandial glucose has been controversial due to low reproducibility and a lack of a standardized method of measurement [32-34]. Postprandial hyperglycemia has been considered as a surrogate of a much more complex series of metabolic events that occur in the postprandial period [33]. These facts support our finding that the relationship between postprandial hyperglycemia and testosterone is confounded by obesity or MetS, or can be explained by an overlap with fasting hyperglycemia or elevated HbA1c. Nonetheless, from a clinical perspective, men with elevated fasting glucose, postprandial glucose, or HbA1c, with or without other metabolic disorder, should be tested for TD.

Total testosterone is composed of free (2-3%), albumin-bound (20–40%), and SHBG-bound testosterone (60-80%). While the present study found that prediabetes was significantly associated with subnormal total testosterone, its association with free or bioavailable testosterone was not significant. This finding is supported by most but not all of the previous studies. In the Rancho Bernardo Study, the total but not the bioavailable testosterone concentration correlated with the insulin resistance and the risk of subsequent development of type 2 diabetes in middle-aged men after adjustment for age, BMI, or systolic blood pressure [5]. Similar results were also observed in two other cohort studies [7,35], in both of which total testosterone, but not free testosterone, was associated with the incidence of diabetes. There is also evidence that the relationship between free testosterone and diabetes is confounded by total body fat or abdominal fat [9,13]. Similarly, we also found that the relationship between prediabetes and free testosterone was confounded by general or central obesity. The finding that prediabetes or diabetes is significantly associated with total testosterone but not free testosterone may be explained by a mechanism associated with SHBG. SHBG has been traditionally considered a protein which binds and transports sex steroids and regulates circulating concentrations of free-form hormones. However, recent studies showed that low SHBG can predict the future development of type 2 diabetes, independent of the testosterone concentration [7,35]. And there is also evidence that mechanisms associated with the SHBG gene is involved in the pathogenesis of type 2 diabetes [36]. As SHBG tightly binds testosterone, which constitutes the largest portion of total testosterone, the low total testosterone in prediabetic or diabetic patients may be simply the consequence of low SHBG. On the other hand, the low SHBG also cause a shift of testosterone from SHBG-bound to free form, which relatively preserves the amount of free testosterone. While the exact mechanism may be complex and involve more factors, the SHBG level, at least in part, explains the discrepant levels of total testosterone and free testosterone in men with prediabetes or diabetes.

The identification of TD in men with prediabetes may have clinical implications. Testosterone replacement therapy (TRT) in hypogonadal men with type 2 diabetes and/or metabolic syndrome improves insulin sensitivity in the short-term [37-40]. It may be of interest to investigate whether hypogonadal men with prediabetes could also benefit from TRT, in terms of improving glucose control and, in a long-term perspective, preventing the future development of overt diabetes. Furthermore, as it has been shown that both prediabetes and low testosterone are independent risk factors for cardiovascular disease and event, and all-cause mortality [41,42], it would be interesting to evaluate whether TRT could decrease the risk of cardiovascular events and mortality in men with both TD and prediabetes. However, it should be noted that the current consensus on the guideline is that TRT be considered only in men with both TD and clinical symptoms [43]. In this way, defined by a combination of total testosterone of <300 ng/dL and Aging Males’ Symptoms (AMS) scale >=27, 14.7% of the prediabetic men in our dataset (unpublished data) could be considered candidates for TRT.

## Conclusions

Men with prediabetes are at an increased risk of subnormal total testosterone, but not free testosterone. The risk is reduced, but remains significant after adjustment for BMI, waist circumference, the number of MetS components, or MetS. After adjustment for MetS, the risk for TD in men with prediabetes is almost equal to that of men with diabetes. The substantially increased risk suggests that testosterone should be measured routinely in men with prediabetes.
