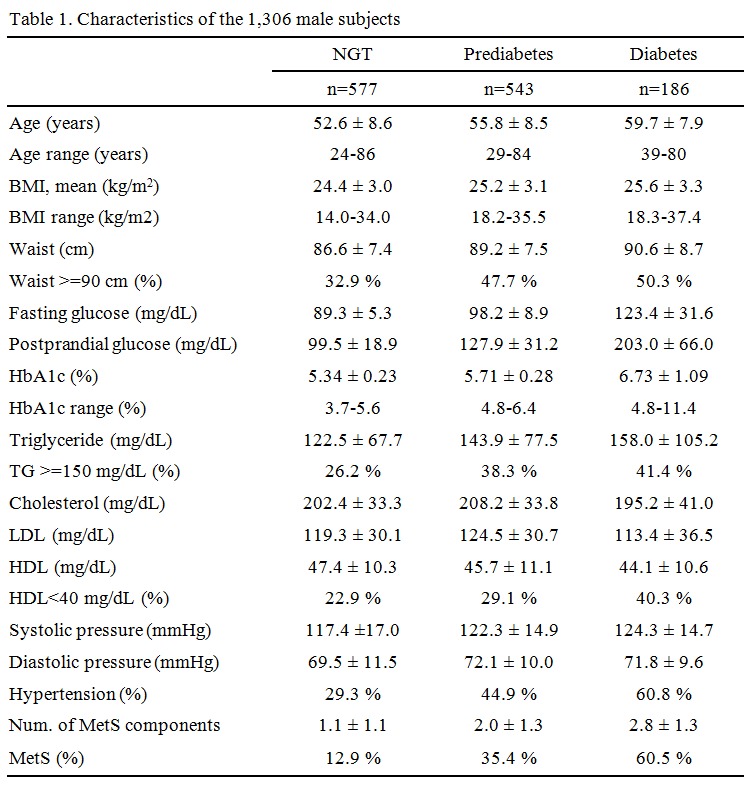# Correction: Prediabetes Is Associated with an Increased Risk of Testosterone Deficiency, Independent of Obesity and Metabolic Syndrome

**DOI:** 10.1371/annotation/c226aa64-8d3b-4c29-971b-84f87b618291

**Published:** 2013-12-13

**Authors:** Chen-Hsun Ho, Hong-Jeng Yu, Chih-Yuan Wang, Fu-Shan Jaw, Ju-Ton Hsieh, Wan-Chung Liao, Yeong-Shiau Pu, Shih-Ping Liu

The values for the rows "Age range (years)", "BMI range (kg/m^2^)", and "HbA1c range (%)" are incorrect. Please see the corrected Table 1 here: 

**Figure:**